# Linked Mutations in the Ebola Virus Polymerase Are Associated with Organ Specific Phenotypes

**DOI:** 10.1128/spectrum.04154-22

**Published:** 2023-03-22

**Authors:** Xiaofeng Dong, Julia Tree, Logan Banadyga, Shihua He, Wenjun Zhu, Tom Tipton, Jade Gouriet, Xiangguo Qiu, Michael J. Elmore, Yper Hall, Miles Carroll, Julian A. Hiscox

**Affiliations:** a Institute of Infection, Veterinary and Ecological Sciences, University of Liverpool, Liverpool, United Kingdom; b UK-Health Security Agency, Salisbury, United Kingdom; c Special Pathogens Program, National Microbiology Laboratory, Public Health Agency of Canada, Winnipeg, Canada; d Wellcome Centre for Human Genetics, Nuffield Department of Medicine, Oxford University, Oxford, United Kingdom; e Pandemic Sciences Institute, Nuffield Department of Medicine, Oxford University, Oxford, United Kingdom; f Infectious Diseases Horizontal Technology Centre (ID HTC), A*STAR, Singapore, Singapore; US Food and Drug Administration

**Keywords:** adaptive mutations, bioinformatics, Ebola virus

## Abstract

Ebola virus (EBOV) causes a severe infection called Ebola virus disease (EVD). The pathogenesis of EBOV infection is complex, and outcome has been associated with a variety of immunological and cellular factors. Disease can result from several mechanisms, including direct organ and endothelial cell damage as a result of viral replication. During the2013 to 2016 Western Africa EBOV outbreak, several mutants emerged, with changes in the genes of nucleoprotein (NP), glycoprotein (GP), and the large (L) protein. Reverse genetic analysis has been used to investigate whether these mutations played any role in pathogenesis with mixed results depending on the experimental system used. Previous studies investigated the impact of three single nonsynonymous mutations (GP-A82V, NP-R111C, and L-D759G) on the fatality rate of mouse and ferret models and suggested that the L-D759G mutation decreased the virulence of EBOV. In this study, the effect of these three mutations was further evaluated by deep sequencing to determine viral population genetics and the host response in longitudinal samples of blood, liver, kidney, spleen, and lung tissues taken from the previous ferret model. The data indicated that the mutations were maintained in the different tissues, but the frequency of minor genomic mutations were different. In addition, compared to wild-type virus, the recombinant mutants had different within host effects, where the D759G (and accompanying Q986H) substitution in the L protein resulted in an upregulation of the immune response in the kidney, liver, spleen, and lungs. Together these studies provide insights into the biology of EBOV mutants both between and within hosts.

**IMPORTANCE** Ebola virus infection can have dramatic effects on the human body which manifest in Ebola virus disease. The outcome of infection is either survival or death and in the former group with the potential of longer-term health consequences and persistent infection. Disease severity is undoubtedly associated with the host response, often with overt inflammatory responses correlated with poorer outcomes. The scale of the2013 to 2016 Western African Ebola virus outbreak revealed new aspects of viral biology. This included the emergence of mutants with potentially altered virulence. Biobanked tissue from ferret models of EBOV infected with different mutants that emerged in the Western Africa outbreak was used to investigate the effect of EBOV genomic variation in different tissues. Overall, the work provided insights into the population genetics of EBOV and showed that different organs in an animal model can respond differently to variants of EBOV.

## INTRODUCTION

Ebola virus (EBOV) causes a devastating infection known as Ebola virus disease (EVD) that in many patients leads to a fatal outcome. The interplay between EVD and outcome is complex and involves a balance between viral load and the host response. Viral load measured as Ct can give some indication for outcome (1, 2), and has been particularly useful in the triage of patients for experimental antiviral trials for EBOV (3). Analysis of samples from patients with EVD treated in Guinea from the 2013 to 2016 outbreak also revealed an immune component that influenced survival (4, 5). Transcriptomic analysis of blood samples from patients in this outbreak demonstrated that individuals who succumbed to the disease showed stronger upregulation of interferon signaling and acute phase responses compared to survivors during the acute phase of infection (6). Particularly notable was the strong upregulation of albumin and fibrinogen genes, which suggested significant liver pathology (6). Cell subtype prediction using mRNA expression patterns indicated that NK-cell populations were increased in patients in the acute phase who survived versus those who died (6), and this was reflected in characterization of the T-cell response (4). In nonhuman primate models of fatal EBOV infection, an extreme aberrant immunological status and anti-inflammatory response was shown to contribute to the development of fatal haemorrhagic fever, and this has also been observed together with lymphocyte death in fatal human infections with EBOV (7).

During replication viral population genetics can be identified by sequencing, this is used to identify a dominant viral genome sequence and minor genomic mutants. Similar to other viruses with RNA genomes, the fidelity of EBOV RNA genome replication and RNA synthesis is influenced by the processivity of the L protein (the viral RNA dependent RNA polymerase [RdRp]) (8). Additionally, there exist potential genome modifications resulting from nucleotide changes by the action of cellular proteins involved in RNA processing, including Adenosine Deaminases Acting on RNA (ADARs) (inducing an A to G transition) (1, 9). The high nucleotide substitution rate can drive the selection of genotypic and phenotypic changes of EBOV that allows the virus to occupy new niches (1). This is evidenced in the ability of EBOV to gain virulence in a small animal model to which it is not initially adapted to replicate (e.g., 10). Concomitant adaptations in viral proteins, such as VP24, were present as minor genomic mutations before they became established as dominant viral genome sequence (10).

Analysis of EBOV population genetics in patients from the 2013 to 2016 Western Africa outbreak showed nonsynonymous differences in minor genomic mutations that had implications for overall viral protein function (11). The greatest frequency of substitution was identified at three codon sites in the L gene (11). The observation of the attenuating N572S, Q986R, and F2061S substitutions in the EBOV L protein was associated with reduced polymerase activity in the context of a mini-replicon system and correlated with lower viral load in patients who survived (11).

Changes in the genotype and phenotype of EBOV were identified over the course of the 2013 to 2016 Western Africa outbreak and there may also be organ specific changes associated with persistence. There have been several documented cases of potential persistence associated with recrudescence in EBOV infection, including in the testes (12), ocular fluid (13), and the central nervous system (14). These observations are also reflected in animal models of persistent asymptomatic infection with EBOV (15). In a ferret model of EVD, virus can be identified in the eyes and there are specific genotypic changes associated with the virus in this organ (16). Analysis of an EVD survivor who suffered a recrudescent infection 10 months after their initial infection indicated that EBOV had entered a period of reduced or minimal replication (17). Mutations with phenotypic consequences of reducing viral replication may be a mechanism that results in persistence and potentially this could be associated with specific organs within an animal.

During the Western Africa outbreak several mutations emerged in EBOV and became dominant. The A82V substitution was located at the NPC1-binding site in GP, the R111C substitution in NP and the D759G substitution in the L protein. Several studies suggested the A82V change was associated with adaptation to growing in primates (18), enhanced infectivity in human (19) and primate cells (18), and improved viral entry (20). Reverse genetics indicated that the D759G substitution in the L protein increased viral transcription and replication and the R111C substitution in NP had the opposite effect (20). In cell culture models, results were varied. Comparison of a virus with these substitutions, made using reverse genetics, compared to one without found that these substitutions conferred a growth advantage in cell culture (20). Separate analysis also found that the NP and L mutants had a replication advantage in cell culture, but this may have been cell type specific (21). In animal models, viruses containing these substitutions did not behave differently to earlier isolates in either a mouse or nonhuman primate model of infection (22). The earlier isolates also displayed higher fitness in cell culture than later isolates with the substitutions (22). In contrast, data from mouse and ferret models suggested that the L mutation decreased the virulence of EBOV (21).

To investigate the relationship between EBOV genotype and phenotype and organ specificity, sequence analysis was carried out on RNA that had been extracted from the organs of domestic ferrets infected with recombinant viruses expressing either the R111C, A82V, or D759G substitutions as part of a previous study (21). The sequence analysis encompassed both virus population genetics and the host response. The data indicated a link between mutation in the viral genome and the host response in each organ from animals infected with mutant and wild-type viruses.

## RESULTS

### Population genetics of the recombinant viruses in blood and organs.

Previous data indicated that the ratio of substitutions between dominant viral genome sequence and minor genomic mutants could influence the activity of EBOV proteins and the outcome of infection (11). This was investigated at a tissue specific level using samples from domestic ferrets that had previously been infected with EBOV/C07 (Wt), EBOV/C07-A82V/GP (G82) mutant, EBOV/C07-R111C/NP (N111) mutant, and EBOV/C07-D759G/L (L759) as described (21). RNA sequencing was used to determine viral sequence and population genetics and identify and quantify host RNA in different tissues.

Tissues and bloods samples were collected from 24 male and female domestic ferrets in eight cages infected with one of the viruses: EBOV/C07 (Wt), EBOV/C07-A82V/GP (G82) mutant, EBOV/C07-R111C/NP (N111) mutant, and EBOV/C07-D759G/L (L759) mutant (Table S1). Based on the quality of total RNA, all 96 samples from the liver, kidney, spleen, and lung tissues of these animals on the succumbing day were sequenced (Table S2). In addition, 72 blood samples taken longitudinally from each of the experimental groups were also sequenced (Table S3).

**FIG 1:**
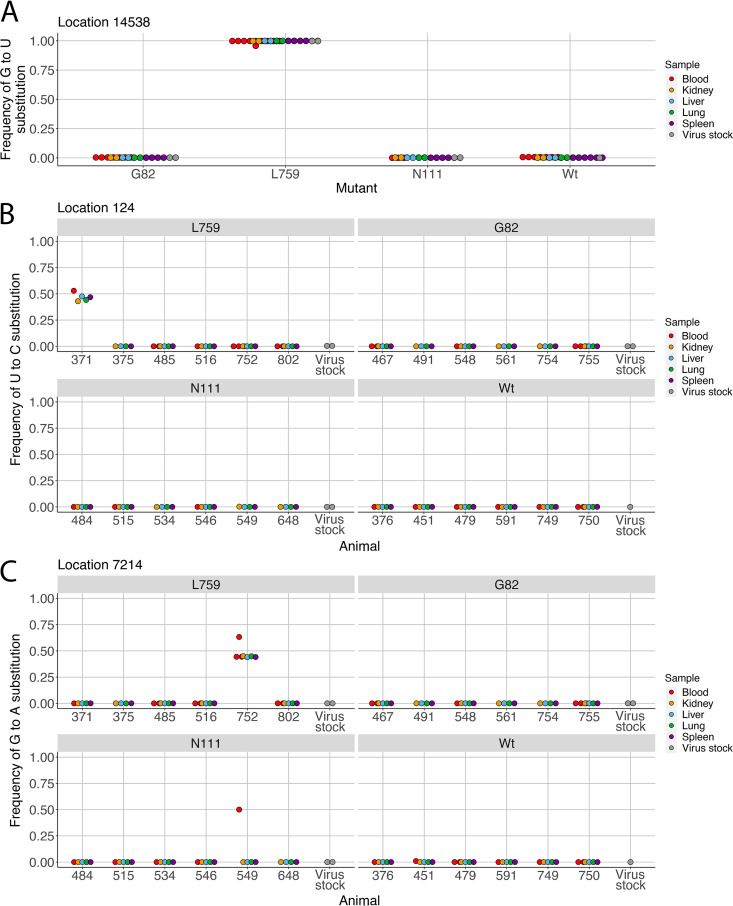
Substitution frequencies of (A) G to U, (B) U to C, and (C) G to A at the genomic positions of 14538, 124, and 7214 in the L759, G82, N111, and Wt EBOV stocks and infected tissues and blood samples.

The dominant viral genome from each sample was generated to identify the nucleotide changes of EBOV in the organ and blood samples by comparing to the EBOV/C07 parental (wild type, Wt) genome (GenBank accession no. KJ660347.2). All reverse engineered substitutions - N111 (location 800), G82 (location 6283), and L759 (location 13856), were identified in the appropriate samples (Tables 1 and 2). Other than the engineered substitutions, four additional nonsynonymous substitutions were identified; VP24-K239Q, L-Q986H, L-I1614T, and L-N1779S (Tables 1 and 2). VP24-K239Q was identified in EBOV sequenced from all organ and blood samples, while L-I1614T and L-N1779S were restricted to an animal infected with the Wt and G82 mutant viruses, respectively (Tables 1 and 2). Interestingly, all EBOV sequenced from organ and blood samples infected with the L759 mutant were accompanied by a L-Q986H (location 14538) change. Substitution of G to U resulting in L-Q986H were found at a frequency of ~100% in both the inoculum and in the domestic ferrets infected with the L759 mutant. However, this change was not found in the inoculum and also in domestic ferrets infected with Wt and other mutants (Fig. 1A; Table S4).

**TABLE 1:** Nucleotide changes (site coverage ≥10) in consensus genome of organ samples by comparing to EBOV/C07 (Wt) EBOV genome

Mutant inoculum	Location	Genes	nt change	nt in inoculum	Codon change	AA change	Animal Ids	Samples under mutant and animal Id
G82	6283	GP	C to U	U	GCG to GTG	82 A to V	All	ST13, ST14, ST15, ST16, ST17, ST18, ST19, ST20, ST1, ST2, ST3, ST4, ST5, ST6, ST7, ST8, ST9, ST10, ST11, ST12, ST21, ST22, ST23, ST24
	10326	Noncoding VP24-VP30	C to U	C	-	-	548	ST17, ST18, ST19, ST20
	12369	L	U to C	U	GTT to GCC	263 V to V	561	ST9, ST10, ST11, ST12
	16916	L	A to G	A	AAT to AGT	1779 N to S	754	ST13, ST14, ST15, ST16
N111	321	5′ UTR	A to C	A	-	-	549	ST61, ST62, ST63, ST64
	800	NP	C to U	U	CGT to TGT	111 R to C	All	ST61, ST62, ST63, ST64, ST65, ST66, ST67, ST68, ST69, ST70, ST71, ST72, ST49, ST50, ST51, ST52, ST53, ST54, ST55, ST56, ST57, ST58, ST59, ST60
L759	13856	L	A to G	G	GAC to GGC	759 D to G	All	ST25, ST26, ST27, ST28, ST29, ST30, ST31, ST32, ST33, ST34, ST35, ST36, ST37, ST38, ST39, ST40, ST41, ST42, ST43, ST44, ST45, ST46, ST47, ST48
	14538	L	G to U	U	CAG to CAT	986 Q to H	All	ST25, ST26, ST27, ST28, ST29, ST30, ST31, ST32, ST33, ST34, ST35, ST36, ST37, ST38, ST39, ST40, ST41, ST42, ST43, ST44, ST45, ST46, ST47, ST48
Wt	121	5′ UTR	U to G	U	-	-	376	ST73, ST74, ST75, ST76
	5366	VP40	C to U	C	GAC to GAT	296 D to D	591	ST93, ST94, ST95, ST96
	16421	L	U to C	U	ATA to ACA	1614 I to T	749	ST85, ST86, ST87, ST88
All	2293	NP	G to U	G	GCC to TCC	608 A to A	All	All
	9267	VP30	C to U	C	AGC to AGT	253 S to S	All	All
	11059	VP24	A to C	A	AAA to CAA	239 K to Q	All	All

**TABLE 2:** Nucleotide changes (site coverage ≥10) in consensus genome of blood samples by comparing to EBOV/C07 (Wt) EBOV genome

Mutant inoculum	Location	Genes	nt change	nt in inoculum	Codon change	AA change	Animal Id	Samples under mutant and animal Id	Notes
G82	6283	GP	C to U	U	GCG to GTG	82 A to V	all	SB7 (day5), SB3 (day5), SB11 (day7), SB4 (day7), SB12 (TER), SB13 (TER)	
	10326	VP24 noncoding	C to U	C			548	64 (day5)	Other samples were not sequenced at this site
	12369	L	U to C	U	GTT to GCC	263 V to V	561	-	Animal 561 was not sequenced at this site
	16916	L	A to G	A	AAT to AGT	1779 N to S	754	-	SB9 (day5) has not this mutation, other samples not sequenced at this site
N111	321	NP noncoding	A to C	A			549	-	Animal 549 was not sequenced at this site
	800	NP	C to U	U	CGT to TGT	111 R to C	All	SB42 (day5), SB45 (day5), SB48 (day5), SB50 (day5)	Other samples were not sequenced at this site
L759	124	NP noncoding	U to C	U			371	SB17 (day7)	Other samples were not sequenced at this site
	7214	GP	G to A	G	GTG to ATG	393 V to M	752	SB35 (day9)	Not found in day7, other samples were not sequenced at this site
	13856	L	A to G	G	GAC to GGC	759 D to G	All	SB17 (day7), SB24 (day7), SB29 (day7), SB33 (day7), SB39 (TER), SB34 (TER), SB25 (TER), SB35 (day9), SB40 (day7)	Other samples were not sequenced at this site
	14538	L	G to U	U	CAG to CAT	986 Q to H	All	SB17 (day7), SB24 (day7), SB29 (day7), SB33 (day7), SB39 (TER), SB34 (TER), SB25 (TER), SB35 (day9), SB40 (day7)	Other samples were not sequenced at this site
Wt	121	NP noncoding	U to G	U			376	SB58 (day5)	Other samples were not sequenced at this site
	5366	VP40	C to U	C	GAC to GAT	296 D to D	591	-	Animal 591 was not sequenced at this site
	16421	L	U to C	U	ATA to ACA	1614 I to T	749	SB68 (day5)	Other samples were not sequenced at this site
All	2293	NP	G to T	G	GCC to TCC	608 A to A	All	All	-
	9267	VP30	C to T	C	AGC to AGT	253 S to S	All	All	-
	11059	VP24	A to C	A	AAA to CAA	239 K to Q	All	All	-

The organ and sequenced blood samples showed almost the same nucleotide changes in the dominant viral genome, except genome position 124 in animal ID 371 and genome position 7214 in animal ID 752 that were unique for a blood sample (Tables 1 and 2). However, these nucleotide changes were also found in other sequenced organ and blood samples from the same animals, and in these cases were the second most frequent nucleotide change (Fig. 1B and C; Tables S5 and 6). These minor changes were animal specific just like the dominant nucleotide changes at genome positions 121, 321, 5366, 10326, 12369, 16421, and 16916 (Fig. 1B and C; Table 1 and 2).

### Minor amino acid variation in EBOV in the organs of ferrets.

**FIG 2:**
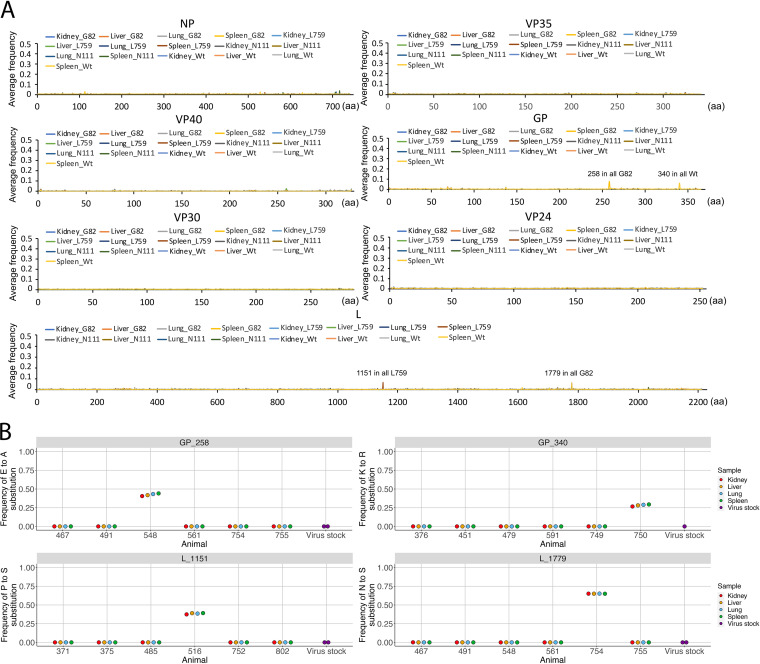
(A) Average amino acid minor variation frequencies for virus infected tissues. (B) Amino acid minor variation frequencies for each tissue samples at GP258, GP340, L1151, and L1779.

**FIG 3:**
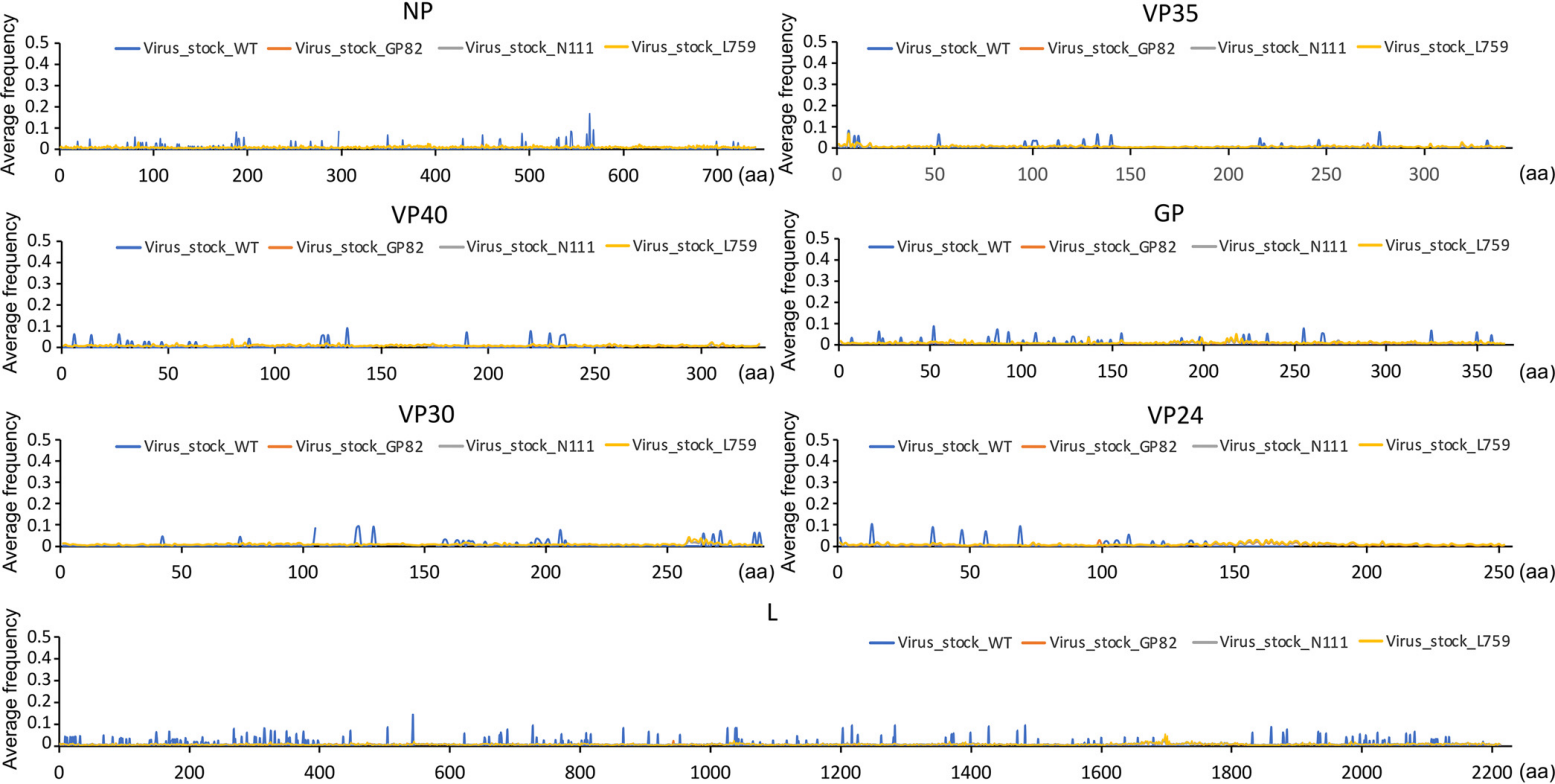
Average amino acid minor variation frequencies for virus stocks. Shown is each gene for EBOV and amino acid position on the *x* axis, and the *y* axis is the frequency of that substitution. The particular virus stock is indicated in the key for each panel.

The minor variation of an amino acid is defined as the nondominant amino acid sequence at that particular site in a protein. This is determined by the ratio of dominant viral genome sequence to minor genomic mutations. Analysis of the minor variation of an amino acid in domestic ferrets infected with either Wt or mutant viruses showed very flat curves (little variation) along the EBOV genome, with only four sites standing out at GP258, GP340, L1151, and L1779 with > 5% average minor variation frequency (Fig. 2A). The most frequent amino acid substitutions at these four sites indicated the minor variation frequencies were raised by a high frequency minor variation of GP-E258A in animal 548 infected with the G82 mutant, GP-K340R in animal 750 infected with Wt, and L-P1151S infected with L759 mutant, a high frequency major variation L-N1779S in animal 754 infected with G82 mutant (Fig. 2B; Table S7). The minor variations of these amino acids were also screened in the virus stock, and none were identified (Fig. 2B; Table S7). Sequencing of the Wt virus stock showed this was more genetically variable than the stock for the three recombinant viruses (Fig. 3). Reflecting the clonal origin of the reverse genetic derived viruses compared to a virus obtained through multiple passage in culture.

### Tissue specific host transcription profiles for the different recombinant viruses.

**FIG 4:**
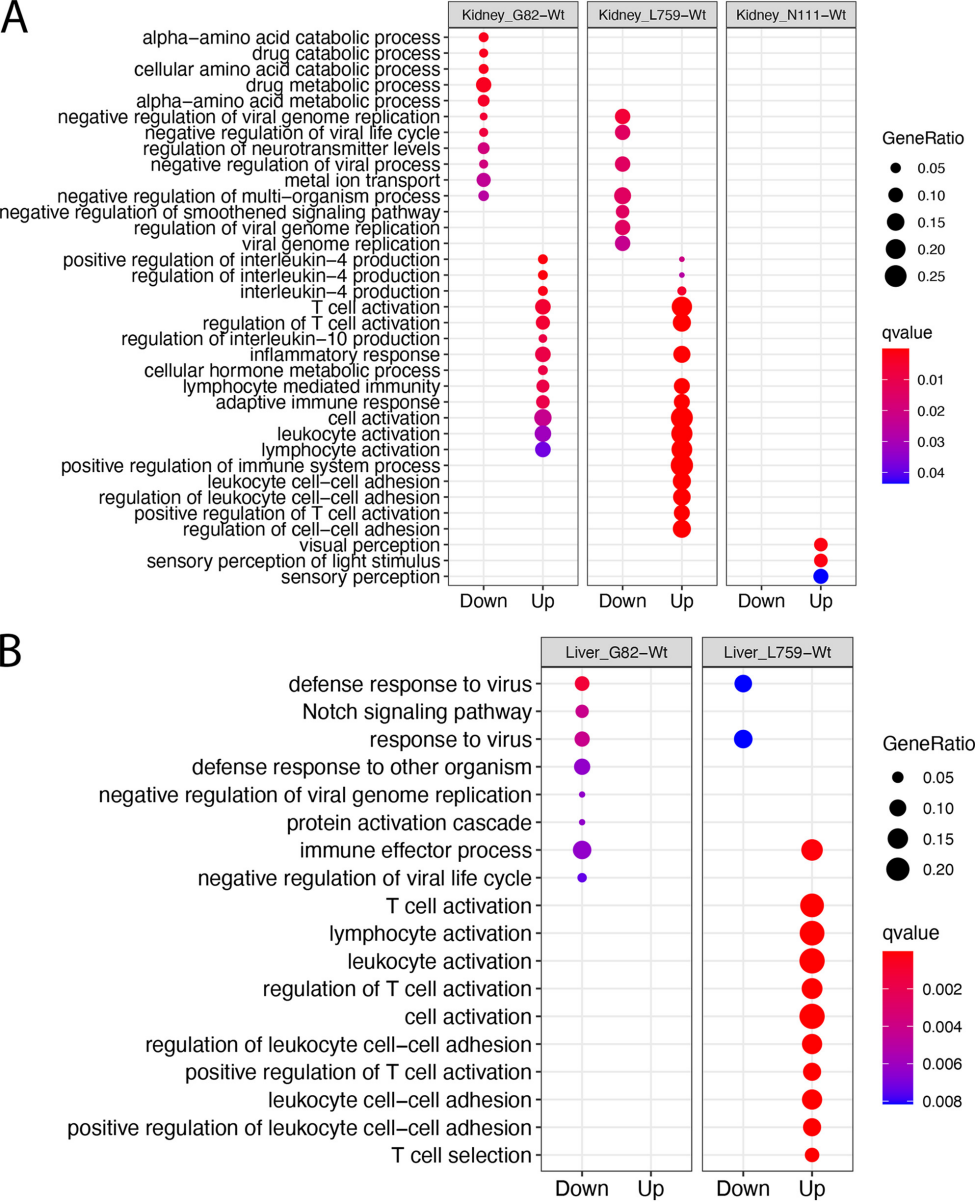
GO enrichment of differentially expressed genes between domestic ferrets infected with either the mutant EBOVs or Wt for (A) Kidneys and (B) Liver.

**FIG 5:**
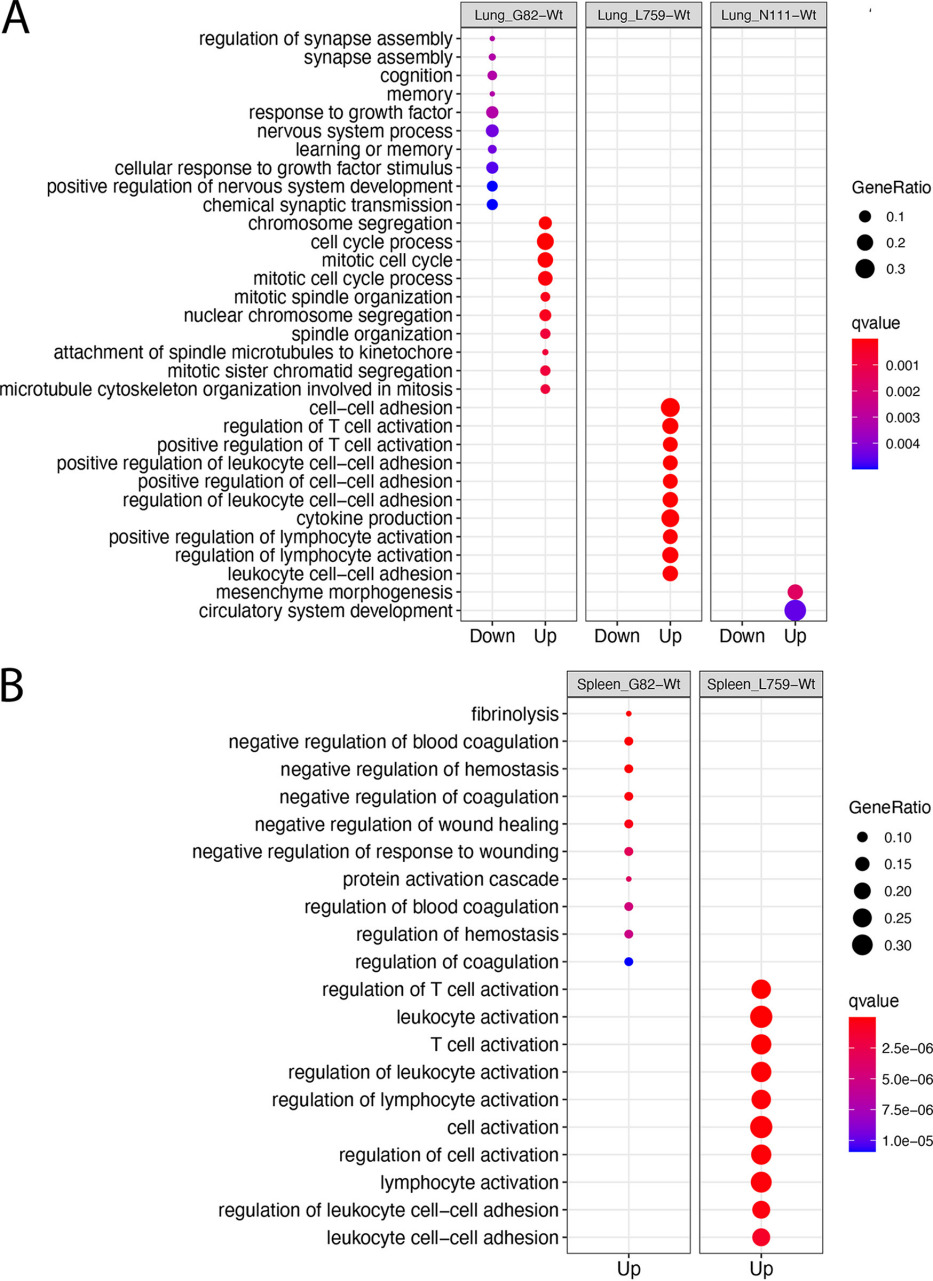
GO enrichment of differentially expressed genes between EBOV mutants and Wt for (A) Lungs and (B) Spleen in the ferret model.

**FIG 6:**
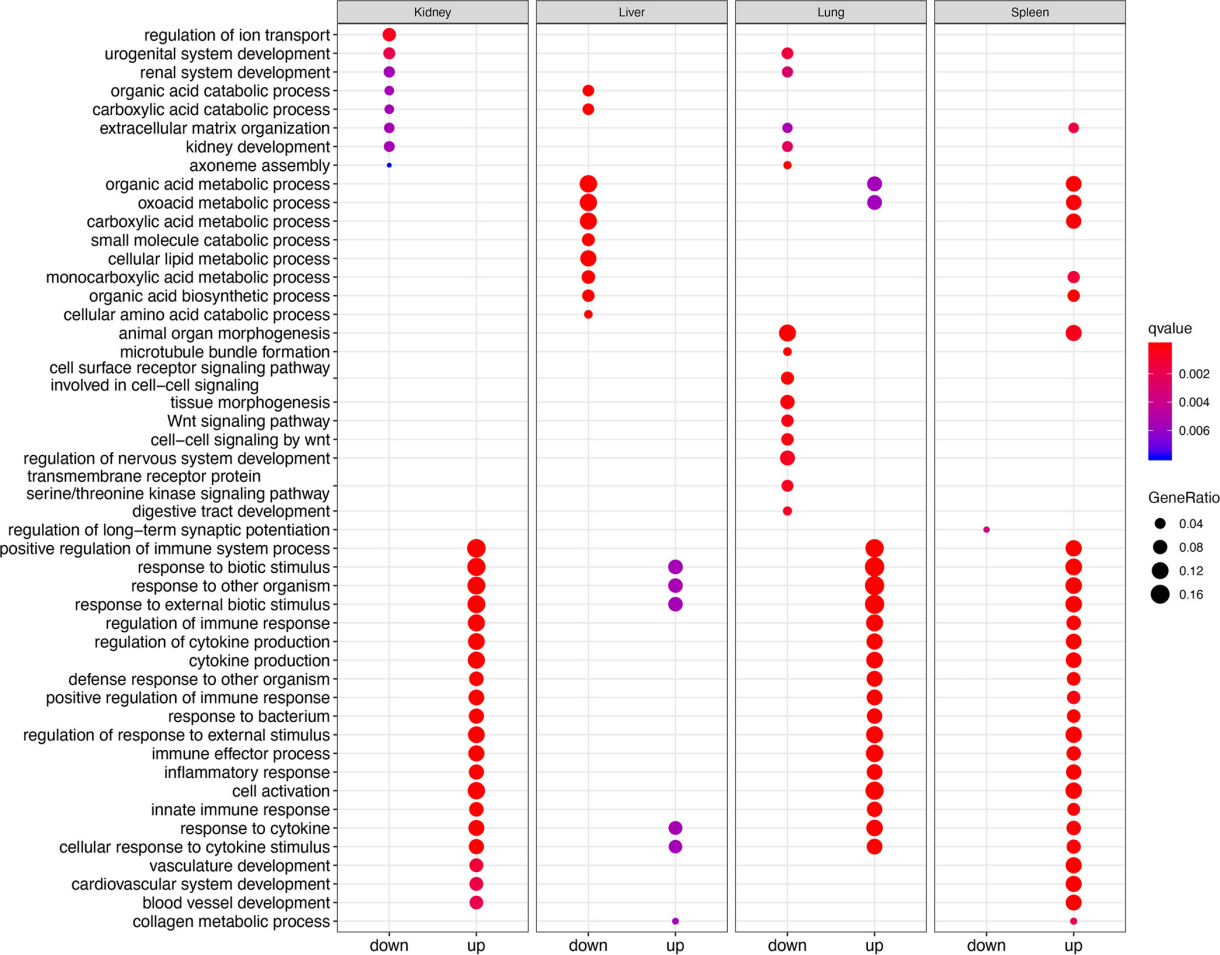
GO enrichment of differentially expressed genes between virus (all mutants and Wt) infected tissues and uninfected controls.

Previously, qRT-PCR analysis had been used to measure viral load in these samples (21). This was interpreted to indicate that the L759 mutant grew less in the domestic ferrets compared to infection with the Wt and other recombinant viruses. The domestic ferrets had delayed and milder weight loss, no noticeable fever, lower clinical scores by day 7, a 2-day delay in some hematological and biochemical parameters, and significantly delayed time to death (48 h) relative to that for Wt, GP mutant, and NP mutant infected animals (21). To investigate whether there was a difference in virulence between the recombinant viruses and Wt virus in terms of the host transcriptomic response, a matrix model design, i.e., (Mutant-control)-(Wt-control), was developed. This was used to identify the genes in each organ responding differently to each mutant compared to the Wt virus (Table S8 to 19). GO enrichment analysis of the data revealed the upregulated genes in organs from domestic ferrets infected with the L759 mutant were significantly enriched in biological processes of T cell activation and leukocyte activation in comparison with domestic ferrets infected with Wt in all four organs (Fig. 4A and B; Fig. 5A and B). Relatively few upregulated genes in the liver (Table S20) were enriched to GO terms relating to the immune response (Fig. 6), perhaps accounting for the previous finding that virus was found to be most abundant in the liver (21).

### Genes responding to time series.

**FIG 7:**
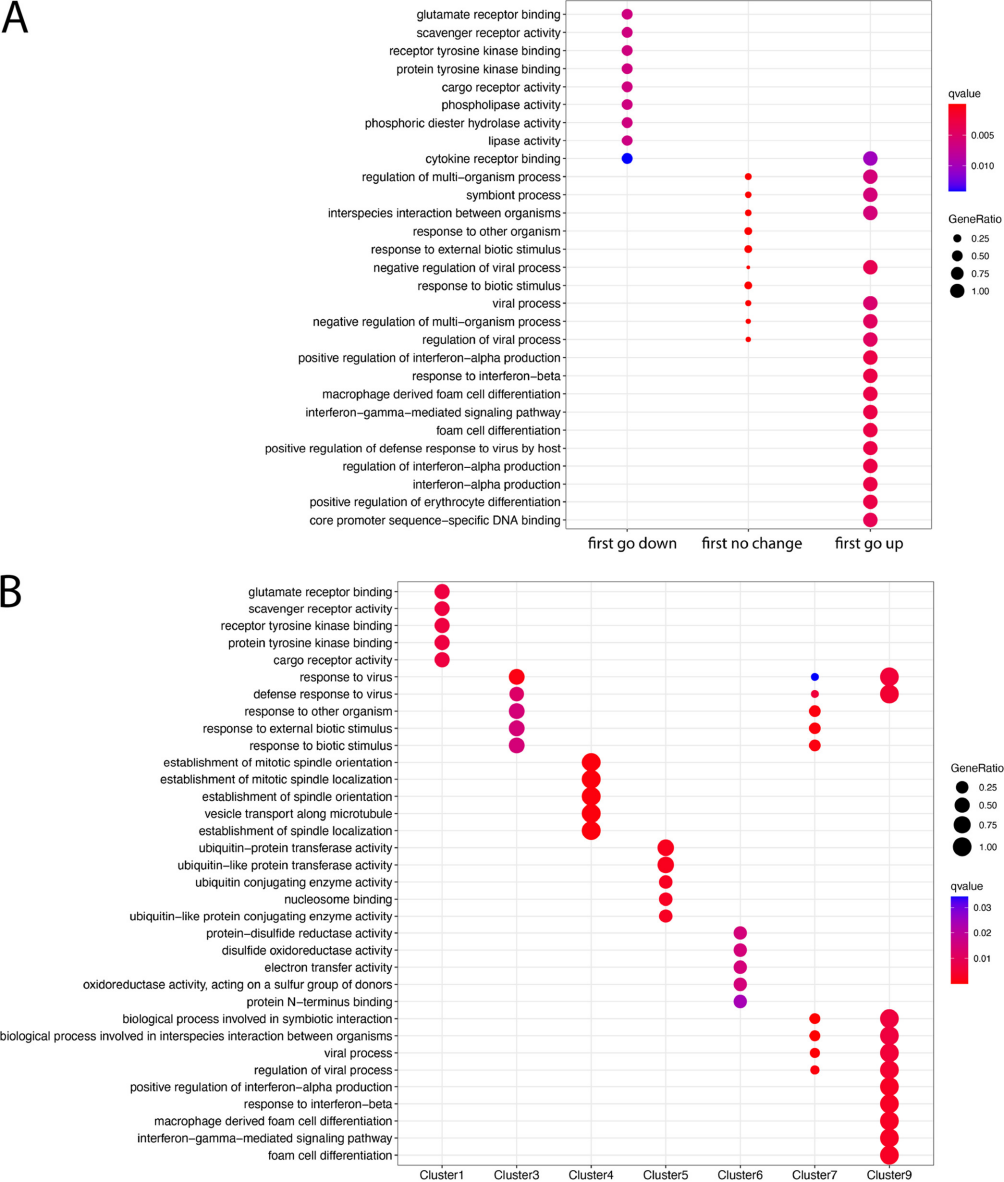
GO enrichment of the dynamic genes clustered by (A) Initial trendy the first segment (B) all segment trends and break points in Table S21.

To study the direction of dynamic changes at gene expression in a time series for the ferret blood samples, the R package Trendy (23) was used. Trendy identified 174 dynamic genes by analyzing the break points that segmented trends in gene expression profiles across four time points (day 0, day 3, day 5, and day 7) (Table S21). The dynamic changes of gene expression correlated with the biological processes of virus infection. The initial trend in abundance in the first segment (trend before the first breakpoint) can be assigned to genes with a direction of “first go up,” “first go down,” or “no change.” There were only four genes which significantly fitted with the initial trend of “first go up” and three genes which significantly fitted with the initial trend of “first go down.” However, 167 genes significantly fitted the initial trend as “no change” (Table S21). GO biological processes enrichment analysis for these three groups of genes revealed the genes assigned to “first go up” and “no change” were directly related to virus defense (Fig. 7A). Dynamic genes with the same expression patterns were further clustered based on initial and later trends of the segments separated by breakpoints (Table S21). We found nine clusters, and cluster 2 to 8 initiated with “first go up.” The GO pathway of “virus defense” was enriched with the genes in clusters 3, 7, and 9 with dynamic patterns of “no change-no change-no change,” “no change-up,” and “up,” respectively (Table S21; Fig. 7B).

### Weighted correlation network analysis indicated there was no relationship between the sex of the animal and disease.

**FIG 8:**
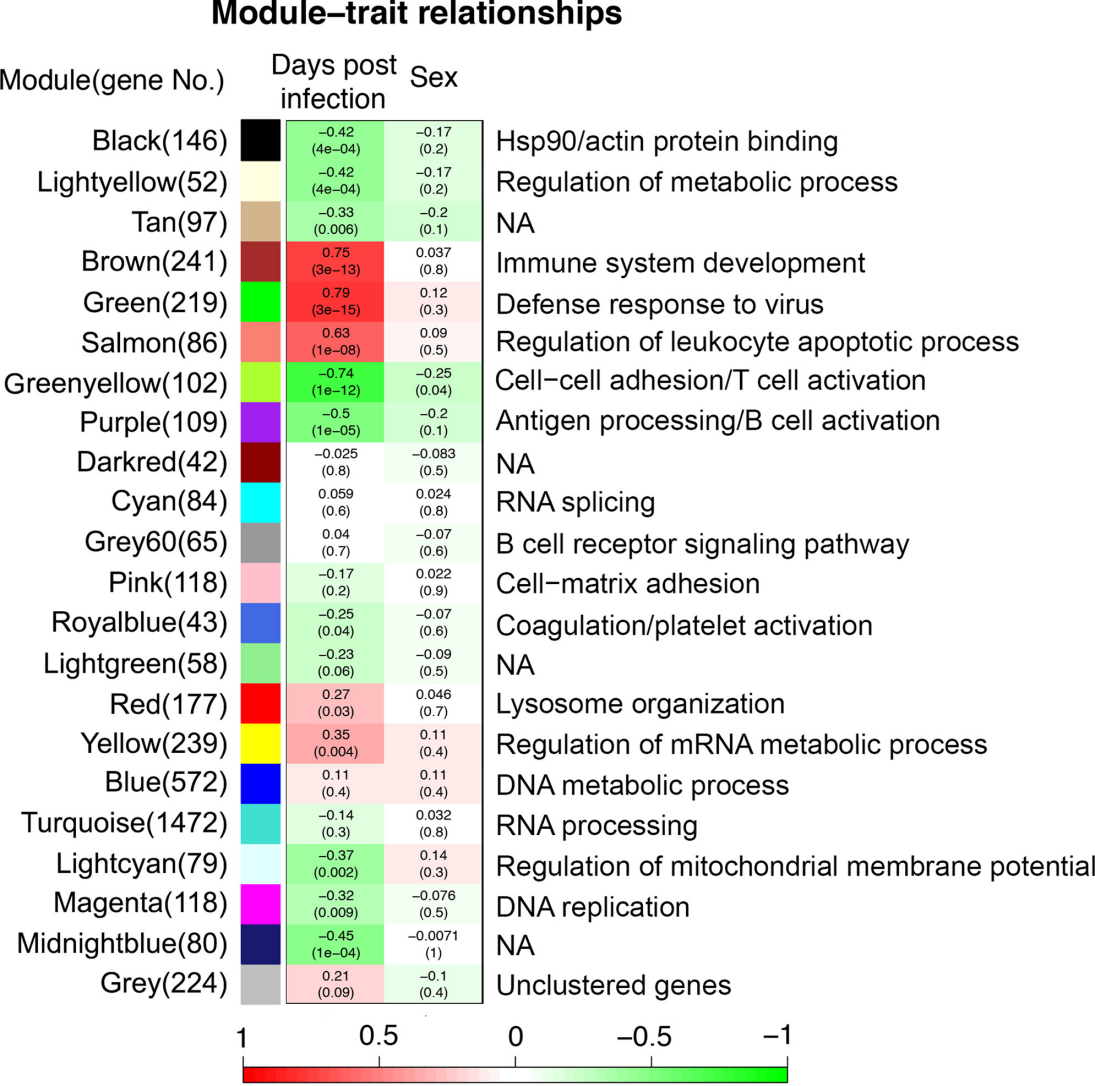
Weighted correlation network analysis (WGCNA) analysis for blood samples. Each color (row) is corresponding to a module and gray module includes all unassigned genes, whereas each column corresponds to a trait (post day of infection and sex). Each cell contains the correlation strength (negatively green/positively red related the traits) and *P*-value (in bracket).

Weighted correlation network analysis (WGCNA) is a systematic biological method that can be used to describe the patterns of gene associating the given traits. This method was applied to explore the relationship between host gene expression and day postinfection and whether the animal was male or female. Highly correlated genes were clustered into the same module based on their gene expression level and shown in different colors. At a general level, the data indicated that the host response was not determined by the sex of the animal, indicating this did not influence virus infection (Fig. 8). The brown module, green module, and salmon module were positively related to virus defense with very strong correlation and *P*-values. This indicated that host gene expression was continuously raised with days of postinfection (Fig. 8). This confirmed that the findings of “first go up” and “no change” were directly related to virus defense. Simultaneously, the genes in the green-yellow module (cell-cell adhesion/T cell activation) and purple module (antigen processing/B cell activation) were strongly downregulated with day postinfection (Fig. 8). Negatively related black module (Hsp90/actin protein binding), light-yellow module (regulation of metabolic process), light-cyan module (regulation of mitochondrial membrane potential), and magenta module (replication). This suggested that potential mechanisms involved in healing collapsed with days postinfection (Fig. 8). A hub gene is the name given to a highly connected gene, located in the module, and these can be candidate biomarkers or potential therapeutic targets (24). Hub genes of the modules with a negative/positive correlation to the days of postinfection trait were identified (Table 3).

**TABLE 3:** Hub gene identified based on the WGCNA analysis

Hub gene	Module	Correlation strength	*P*-value	Gene name	Gene description
ENSMPUG00000022393	Black	−0.42	0.0004	F9	Coagulation factor IX
ENSMPUG00000014498	Light-yellow	−0.42	0.0004	SBF1	SET binding factor 1
ENSMPUG00000002054	Tan	−0.33	0.006	ACOD1	Aconitate decarboxylase 1
ENSMPUG00000012497	Brown	0.75	3E-13	ALAS2	5′-aminolevulinate synthase 2
ENSMPUG00000003233	Green	0.79	3E-15	CD3E	CD3e molecule
ENSMPUG00000010429	Salmon	0.63	0.00000001	ELMO1	Engulfment and cell motility 1
ENSMPUG00000004470	Green-yellow	−0.74	1E-12	ETS1	ETS proto-oncogene 1, transcription factor
ENSMPUG00000008514	Purple	−0.5	0.00001	ITGA2B	Integrin subunit alpha 2b
ENSMPUG00000013696	Dark-red	−0.025	0.8	WDFY3	WD repeat and FYVE domain containing 3
ENSMPUG00000014809	Cyan	0.059	0.6	KIF14	Kinesin family member 14
ENSMPUG00000007802	Grey60	0.04	0.7	ACTR3	Actin related protein 3
ENSMPUG00000001778	Pink	−0.17	0.2	JMJD1C	Jumonji domain containing 1C
ENSMPUG00000009285	Royal-blue	−0.25	0.04	EHD1	EH domain containing 1
ENSMPUG00000011122	Light-green	−0.23	0.06	HPRT1	Hypoxanthine phosphoribosyltransferase 1
ENSMPUG00000010717	Red	0.27	0.03	ANXA6	Annexin A6
ENSMPUG00000014719	Yellow	0.35	0.004	SNX29	Sorting nexin 29
ENSMPUG00000010474	Blue	0.11	0.4	PIK3R1	Phosphoinositide-3-kinase regulatory subunit 1
ENSMPUG00000016843	Turquoise	−0.14	0.3	THRAP3	Thyroid hormone receptor associated protein 3
ENSMPUG00000003302	Light-cyan	−0.37	0.002	CCDC66	Coiled-coil domain containing 66
ENSMPUG00000012699	Magenta	−0.32	0.009	-	-
ENSMPUG00000020859	Midnight-blue	−0.45	0.0001	-	Small nucleolar RNA, C/D box 10

## DISCUSSION

Due to the nature of EBOV infection and EVD, human challenge models are not available to study infection and the action of medical countermeasures. Ferrets have been evaluated as a small animal model for EVD in humans (25). Ferrets reproduce the disease and lethality in humans (16, 26), including longer term sequala such as persistence (16). During the 2013 to 2016 West African outbreak, there were genetic changes in EBOV as the outbreak progressed (1) and at a minor mutation level these may have impacted upon the disease phenotype (11). Several substitutions were identified in the dominant genome sequence including in GP, NP, and the L protein. These changes and how they may have affected virus and host biology had been evaluated in various model systems, including cell culture and animal models with either inconsistent or conflicting outcomes. A previous study in ferrets using recombinant EBOV indicated that the L759 mutant potentially decreased virulence compared to infection with Wt, N111, or G82 mutant (21). Using tissues from this study, we evaluated the effect of these mutations on viral evolution and the host response in individual organs and the blood by an RNA sequencing approach.

Analysis of dominant viral genome sequences from the 2013 to 2016 Western Africa EBOV outbreak suggested the emergence of mutants with potentially altered virulence (21). Organ specific genotypic change identified in virus sampled from the eyes of infected ferrets were associated with potential persistence (16). Hence, we were interested in investigating the viral evolutionary profiles in the organs of ferrets infected with different mutants. We found that some of the dominant nucleotide mutations identified were specific for an individual animal, revealing potential host specific differences when responding to virus infection. The substitution in VP24-K239Q, as a result of an A to C mutation, was widespread in all samples sequenced, and was perhaps a host adapting mutation. Previously, mutations in VP24 have been shown to be involved in this process (10). The G to U mutation which caused a change of L-Q986H was specific to the L759 mutant recombinant virus. We postulate that this amino acid change could be linked to the L-D759G change while the L759 was being in GripTite 293 MSR and VeroE6 cells, prior to being used in the experimental work that was originally described (21). The high site coverage for this amino acid substitution during sequencing eliminated the possibility of sequencing error and/or contamination (Table S4). The virus stocks were prepared from pooled extracts of cell cultures. L-Q986H was found with ~100% variation in the virus stock of L759 and indicated all cell cultures contributed high L-Q986H percentage of L-Q986H to the stock pool.

Prominent minor variations (with higher frequency) in amino acid sequences in EBOV have been identified in infected humans and can be critical in patients with EVD, potentially contributing toward a survival or a fatal outcome (11). In the ferret infection study, four such minor variant genomic changes were identified. However, these were animal specific and not widespread in the stock Wt and mutant viruses. This contrasts with potential minor amino acid variations in EBOV in tissues, such as the eye, where a persistent infection can occur (16). Ferrets are likely to have differing immune selection pressures from humans to promote the emergence or selection of minor variant genomes. In addition, in this experiment, the ferrets were inoculated with relatively pure virus stocks derived from reverse genetic clones (Fig. 3). Therefore, it is likely that the underlying genetic variation in these viruses is less than that would be observed during infection of the virus in the wild, such as found in human-to-human transmission.

Bioinformatic methods of Trendy and WGCNA were used to investigate the dynamic changes of host responses to EBOV with longitudinal blood samples. Trendy identified genes with dynamic changes while WGCNA was used to determine gene modules with dynamic changes. Both methods indicated genes related to virus defense were upregulated with day postinfection. Using WGCNA, we identified Hub genes of the modules with a negative/positive correlation to the days of postinfection. Hub genes are the genes with high correlation in candidate modules, expected to provide potential targets for diagnosis and treatment (27), especially the Hub genes in the virus defense and immune response modules (Fig. 8; Table 3).

The L759 mutant in the L protein was associated with decreased virulence of EBOV, *in vivo*, as observed in mice and ferrets, but the mechanism was not addressed (21). The effect on host biology was evaluated in the different tissue with RNA-seq data. Compared to L759, the other mutants showed less, or no genes enriched in biological processes of T cell activation and leukocyte adhesion in organs (Fig. 4A and B; Fig. 5A and B). This suggested that infection with the L759 mutant led to a stronger immune response than the Wt, G82, or N111 mutants. There was no evidence to suggest that the L-D759G substitution significantly reduced the virulence of EBOV in humans or animals. However, a previous study suggested the minor change of Q to H at L986 could significantly push patients with EVD toward a survival outcome (11). L-D759G linked L-Q986H may contribute mainly to the pathogenesis in L mutant-infected domestic ferrets which is slower than that with the other three viruses. The study indicated that not only can amino acid substitutions in the EBOV genome manifest in different disease profiles, these changes also more subtlety affect the host response at an organ specific level.

## MATERIALS AND METHODS

### Sample collection.

Total RNA samples were provided by Public Health Agency of Canada that have been used for the qRT-PCR analysis (21). In brief, eight groups of domestic ferrets (three animals per group) were infected with one of the viruses: EBOV/C07 (Wt), EBOV/C07-A82V/GP mutant, EBOV/C07-R111C/NP mutant, and EBOV/C07-D759G/L mutant (Table S1); the virus stocks were prepared and the viral genome confirmed by next-generation sequencing; Whole-blood samples from all domestic ferrets were collected every day until animals succumbing, then major organs (liver, kidneys, spleen, and lungs) were harvested from all euthanized domestic ferrets (Table S1); total RNA extracted from the ferret whole-blood and organs samples.

### Sample sequencing.

In total, 72 blood samples were sequenced (Table S2). Following the manufactures protocols, where available 100 ng of total RNA was used as input material in to the QIAseq FastSelect –rRNA (HMR) (Qiagen) protocol to remove cytoplasmic and mitochondrial rRNA with a fragmentation time of 7 min. Subsequently the NEBNext Ultra II Directional RNA Library Prep Kit for Illumina (New England Biolabs) was used to generate the RNA libraries. Following 13 cycles of amplification, the libraries were purified using Ampure XP beads. Each library was quantified using Qubit and the size distribution assessed using the Fragment analyzer. These final libraries were pooled in equimolar amounts using the Qubit and Fragment analyzer data. The quantity and quality of the pool was assessed by the Bioanalyzer and subsequently by qPCR using the Illumina Library Quantification Kit from Kapa on a Roche Light Cycler LC480II according to manufacturer's instructions. The template DNA was denatured according to the protocol described in the Illumina cBot User guide and loaded at 300pM concentration. To improve sequencing quality control 1% PhiX was spiked-in. The sequencing was carried out on seven lanes of an Illumina HiSeq 4000 using v3 chemistry, generating 2 × 150 bp paired end reads.

We sequenced 96 organ samples (Table S3). Following the manufactures protocols, 800 ng of total RNA was used as input material in to the QIAseq FastSelect –rRNA (HMR) (Qiagen) protocol to remove cytoplasmic and mitochondrial rRNA with a fragmentation time of 7, 13, or 15 min. Subsequently the NEBNext Ultra II Directional RNA Library Prep Kit for Illumina (New England Biolabs) was used to generate the RNA libraries. Following 8 cycles of amplification the libraries were purified using Ampure XP beads. Each library was quantified using Qubit and the size distribution assessed using the Fragment analyzer. Final libraries were pooled in equimolar amounts using the Qubit and Bioanalyzer data. The quantity and quality of each pool was assessed by Bioanalyzer and subsequently by qPCR using the Illumina Library Quantification Kit from Kapa (KK4854) on a Roche Light Cycler LC480II according to manufacturer's instructions. Briefly, a 10 μL PCR (performed in triplicate for each pooled library) was prepared on ice with 6 μL SYBR green I Master Mix and 2 μL diluted pooled DNA (1:1,000 to 1:100,000 depending on the initial concentration determined by the Qubit dsDNA HS assay kit). PCR thermal cycling conditions consisted of initial denaturation at 95°C for 5 min, 35 cycles of 95°C for 30 s (denaturation) and 60°C for 45 s (annealing and extension), melt curve analysis to 95°C (continuous), and cooling at 37°C (LightCycler LC48011, Roche Diagnostics Ltd., Burgess Hill, UK). Following calculation of the molarity using qPCR data, template DNA was diluted to 300pM and denatured for 8 min at room temperature using freshly diluted 0.2 N sodium hydroxide (NaOH) and the reaction was subsequently terminated by the addition of 400 mM TrisCl ph = 8. To improve sequencing quality control 1% PhiX was spiked-in. The libraries were sequenced on the Illumina NovaSeq 6000 platform following the XP workflow on three lanes of an S4 flow cell, generating 2 × 150 bp paired-end reads.

The raw fastq files generated by Illumina HiSeq and Novaseq were trimmed to remove Illumina adapter sequences using Cutadapt v1.2.1 (28). The option “−O 3” was set, so the that 3′ end of any reads which matched the adapter sequence with greater than 3 bp was trimmed off. The reads were further trimmed to remove low quality bases, using Sickle v1.200 (29) with a minimum window quality score of 20. After trimming, reads shorter than 10 bp were removed.

### Differential expression analysis.

Hisat2 v2.1.0 (30) was used to map the trimmed reads on the *Mustela putorius* reference genome assembly with known splice sites (release-96) downloaded from the Ensembl FTP site. The alignments were used for calculating read counts per gene using featureCounts v2.0.0 (31) with a guide Ensembl GTF (release-96). The raw counts generated from featureCounts were imported into the R v3.6.3 environment to carry out differential expression analysis using edgeR v3.26.8 (32). The design model matrix for edgeR v3.26.8 was described as model.matrix (~Tissue+Tissue:Sex+Tissue:Mutants), where “Tissue:Sex” was used to block the sex effect to find genes that have responded differently to the Wt and the mutants in each organ, and find that have responded differently to the Wt and any mutants in each organ. Differentially expressed genes with an FDR < 0.05 and an absolute log_2_ fold change > 1 were reported. The GO enrichment in biological process for the differentially expressed genes was performed using enrichGO function in clusterProfiler v3.12.0 (33) to query the *Mustela putorius* GO database (object: AH72558) via AnnotationHub v2.16.1. GO terms with q-value < 0.05 were considered significantly enriched and top 10 most significant categories of each cluster was plotted.

### Consensus virus genomes and nucleotide frequencies.

The reads unmapped to the *Mustela putorius* reference genome assembly were extracted by bam2fastq (v1.1.0) and then mapped on the EBOV/C07 (Wt) EBOV genome (GenBank accession no. KJ660347.2) using Bowtie2 v2.3.5.1 (30) by setting the options to parameters “–local -X 2000 –no-mixed,” followed by Sam file to Bam file conversion, sorting, and removal of the reads with a mapping quality score below 11 using SAMtools v1.9 (34). After that, the PCR and optical duplicate reads in the bam files were discarded using the MarkDuplicates in the Picard toolkit v2.18.25 (http://broadinstitute.github.io/picard/) with the option of “REMOVE_DUPLICATES=true.” The resultant Bam file was processed by Quasirecomb v1.2 (35) to generate read coverage of each nucleotide site and a phred-weighted table of nucleotide frequencies. The phred-weighted table was parsed with a custom perl script to generate a dominant genome sequence as our previous description (1). The dominant genome sequence was then used as a template in the second round of mapping to generate a consensus genome (site coverage > 10). With this method, we generated site coverage, nucleotide frequencies and consensus genome sequence of EBOV in all tissue and blood samples.

### Minor variation of amino acid.

The minor variations of amino acid in the genes of virus were called as our previous description (11). Reads (unmapped on human genome) were aligned to the consensus EBOV genome sequence using Bowtie2 with the parameter of “–local -X 2000 –no-mixed.” The Bowtie2 outputs were processed in the same way as above to generate a Bam file without read duplication. This Bam file was then processed by diversiutils script in DiversiTools (http://josephhughes.github.io/btctools/) with the “-orfs” function to generate the number of amino acid change caused by the nucleotide deviation at each site in protein. In order to distinguish of low frequency mutations from Illumina sequence errors, the diversiutils used the calling algorithms based on the Illumina quality scores to calculate a *P*-value for each mutation at each nucleotide site (36). The amino acid change was then filtered based on the *P*-value (<0.05) and amino acid site coverage (>5) to remove the low frequency mutations from Illumina sequence errors.

### Genes responding to time series.

The R package Trendy v1.6.4 (23) was used to identify the dynamic changes at genes in time series (day0, day3, day5 and day7) of sequenced blood samples with default setting. The input normalized count was prepared using Median Normalization via the MedianNorm and GetNormalizedMat functions in the EBSeq v1.24.0 package. Top dynamic genes were extracted the topTrendy function in Trendy package. The GO enrichment in biological process for top dynamic genes was performed using enrichGO function in clusterProfiler v3.12.0 (33) to query the *Mustela putorius* GO database (object: AH72558) via AnnotationHub v2.16.1. GO terms with q-value < 0.05 (adjusted *P*-value cutoff on enrichment tests to prevent high false discovery rate) were considered significantly enriched and top 10 most significant categories of each cluster was plotted. Dots in the plot represent GO term enrichment with color coding: red indicates high enrichment, blue indicates low enrichment. The sizes of the dots represent the ratio of input genes that are annotated in a GO term.

### Weighted correlation network analysis.

Gene-network analysis was performed as our previous description (37). Before performance of gene-network analysis for blood samples, low-expression genes, at least one read per million in 60 (90%) of the samples, were filtered and then gene counts were normalized and log-transformed with the command rpkm() in edgeR v3.26.8. The input normalized count was prepared using rlog functions with option “blind=TRUE” in the DESeq2 v1.24.0 package. All sequenced samples blood samples (Table S2), except the terminal bleeds (TER), were used for WGCNA analysis, and no outgroups were found by R function hclust in these samples. Then 21 modules were derived from WGCNA v1.70.3 (24) with blockwiseConsensusModules function (power = 6, TOMType=“signed,” corType=“bicor,” maxPOutliers = 0.1, minModuleSize = 30, deepSplit = 4). Hub genes in these modules were calculated by the chooseTopHubInEachModule function from the WGCNA package was used to identify the hub genes with default setting.

### Data availability.

Illumina data sets are available under NCBI PRJNA856238.

Bioinformatic codes and dominant viral genome sequences are available at https://github.com/Hiscox-lab/Ebola_linked_mutations.
