# Supplementary material for: Linked Mutations in the Ebola Virus Polymerase Are Associated with Organ Specific Phenotypes
Source: Microbiol Spectr. 2023 Mar 22;11(2):e04154-22. doi: 10.1128/spectrum.04154-22 (PMC10101120; doi:10.1128/spectrum.04154-22)
Supplement: Supplemental file 1 — Legends of Tables S1 to S21. Download spectrum.04154-22-s0001.pdf, PDF file, 0.04 MB [file spectrum.04154-22-s0001.pdf]

Supplementary Table legends

Supplementary Table 1. Information of experiment design.

Supplementary Table 2. Information of sequenced ferret tissue samples.

Supplementary Table 3. Information of sequenced ferret blood samples.

Supplementary Table 4. Nucleotide frequencies and site coverage at the genomic positions of 14538 in the L759, G82, N111 and Wt EBOV stocks and infected tissues and blood samples.

Supplementary Table 5. Nucleotide frequencies and site coverage at the genomic positions of 124 in the L759, G82, N111 and Wt EBOV stocks and infected tissues and blood samples.

Supplementary Table 6. Nucleotide frequencies and site coverage at the genomic positions of 7214 in the L759, G82, N111 and Wt EBOV stocks and infected tissues and blood samples.

Supplementary Table 7. Top three amino acid variation frequencies for each tissue samples and virus stocks at GP258, GP340, L1151 and L1779.

Supplementary Table 8. Differentially expressed genes between domestic ferrets infected with G82 and Wt for Kidneys in Figure 4A.

Supplementary Table 9. Differentially expressed genes between domestic ferrets infected with L759 and Wt for Kidneys in Figure 4A.

Supplementary Table 10. Differentially expressed genes between domestic ferrets infected with N111 and Wt for Kidneys in Figure 4A.

Supplementary Table 11. Differentially expressed genes between domestic ferrets infected with G82 and Wt for Liver in Figure 4B.

Supplementary Table 12. Differentially expressed genes between domestic ferrets infected with L759 and Wt for Liver in Figure 4B.

Supplementary Table 13. Differentially expressed genes between domestic ferrets infected with N111 and Wt for Liver in Figure 4B.

Supplementary Table 14. Differentially expressed genes between domestic ferrets infected with G82 and Wt for Lungs in Figure 5A.

Supplementary Table 15. Differentially expressed genes between domestic ferrets infected with L759 and Wt for Lungs in Figure 5A.

Supplementary Table 16. Differentially expressed genes between domestic ferrets infected with N111 and Wt for Lungs in Figure 5A.

Supplementary Table 17. Differentially expressed genes between domestic ferrets infected with G82 and Wt for Spleen in Figure 5B.

Supplementary Table 18. Differentially expressed genes between domestic ferrets infected with L759 and Wt for Spleen in Figure 5B.

Supplementary Table 19. Differentially expressed genes between domestic ferrets infected with N111 and Wt for Spleen in Figure 5B.

Supplementary Table 20. Differentially expressed genes between virus infected tissues and uninfected controls in Figure 6.

Supplementary Table 21. Dynamic genes with trend of segments separated by breakpoints identified by Trendy.
